# Perceived problematic alcohol use in the family and adolescents’ stress-related complaints: examining the buffering role of the school’s degree of student focus as rated by its teachers

**DOI:** 10.1186/s12889-023-16505-x

**Published:** 2023-09-09

**Authors:** Sara Brolin Låftman, Charlotta Magnusson, Gabriella Olsson, Joakim Wahlström, Bitte Modin

**Affiliations:** 1 Department of Public Health Sciences, Centre for Health Equity Studies (CHESS), Stockholm University, 10691 Stockholm, Sweden; 2 Swedish Institute for Social Research (SOFI), Stockholm University, 10691 Stockholm, Sweden

**Keywords:** Alcohol, Stress, Somatic complaints, Psychological distress, Adolescents, School, Compensatory, Contextual, Multilevel

## Abstract

**Background:**

A non-negligible proportion of children grow up with problematic alcohol use in the family. Problematic familial drinking can be regarded as a stressor, and prior studies have consistently reported poorer mental health among adolescents who are exposed. However, it is also of relevance to identify modifiable protective factors which may buffer against stress-related ill-health in this group of adolescents. One context where such factors may be present is the school. The aim of this study is to examine the relationship between perceived problematic familial alcohol use and students’ stress-related complaints, and specifically to explore if the school’s degree of student focus can buffer against any such negative health consequences of problem drinking at home.

**Methods:**

Data were drawn from four separate surveys, the Stockholm School Survey (SSS) and the Stockholm Teacher Survey (STS) conducted in 2014 and 2016 among 7,944 students (~ 15–16 years) and 2,024 teachers in 147 Stockholm senior-level school units. Perceived problematic familial alcohol use was measured by one item in the SSS. Stress-related complaints were captured by co-occurring somatic complaints and psychological distress, and reported by students in the SSS. The school’s student focus was measured by an index based on teachers’ ratings of four items in the STS. A set of covariates at the student and the school level were also included. Two-level binary logistic and linear regression models were performed.

**Results:**

Perceived problematic familial alcohol use was linked with an increased likelihood of reporting co-occurring somatic complaints as well as psychological distress. Cross-level interactions revealed that the association between perceived problematic familial alcohol use and co-occurring somatic complaints was weaker among students attending schools with stronger teacher-rated student focus. Regarding psychological distress, the association was weaker for students attending schools with intermediate or strong teacher-rated student focus, compared with those attending schools with weaker teacher-rated student focus.

**Conclusions:**

The findings provide support for the assumption that favourable conditions in schools can buffer against negative health consequences of problematic conditions in the family, thus serving a compensatory role.

**Supplementary Information:**

The online version contains supplementary material available at 10.1186/s12889-023-16505-x.

## Background^1^

A non-negligible proportion of children and adolescents are exposed to problematic alcohol use in the family. Studies from the U.S., Denmark, and Sweden have shown that between 4 and 23% have parents with problematic alcohol use, depending on how alcohol problems were measured [2].

Problematic familial drinking can be regarded as a stressor, with potential effects on stress-related ill-health for those who are exposed, not least for the children. Indeed, prior studies have shown problematic parental alcohol use to be associated with mental health problems in the offspring in terms of internalising problems such as emotional symptoms and psychosomatic complaints [3–9], as well as mental and behavioural disorders [10] including self-injury and suicidal behaviour [11–13]. Although some earlier studies have measured problematic parental alcohol use through, e.g., official register information [10], military conscription records [13] or by parental self-reports [7], much research has focused on the adolescents’ own perceptions of problematic parental alcohol use captured by e.g., the Children of Alcoholics Screening Test (CAST) scale [5, 6, 9] or by single items [3, 4, 8, 12].

Problematic parental alcohol use may lead to stress in the children due to negative emotions such as fear, shame and guilt [14–16], impaired relations with parents [3] and the need to take responsibility or parenting roles at an early age [15]. However, as shown by the vast literature on Adverse Childhood Experiences (ACEs), parental problematic alcohol use often coincides with other aspects of household dysfunction, e.g., violence, abuse, neglect, and incarceration [17–19], which may cause, moderate or mediate the associations between parental problematic alcohol use and adverse outcomes.

Two systematic reviews have provided overviews of protective mental health factors in children of parents with alcohol-related disorders. Park & Schepp [20] identified vulnerability and resilience factors at four levels: the individual; parental; familial; and social level. Wlodarczyk et al. [21] classified protective factors into three domains, which they labelled as child-related factors; family and parental factors; and environmental factors. Social support is a key factor that was listed both at the social level by Park & Schepp [20] and in the environmental domain by Wlodarczyk et al. [21]. This may include social support from various sources, e.g., grandparents, parents of friends, and teachers as well [15, 20, 21]. Indeed, the presence of stable adult figures outside the closest family has been highlighted as an important protective factor [15]. Overall, however, it has been highlighted that protective mental health factors in children of parents with alcohol-related disorders is an understudied area [21] and hence there is a need for further inquiry. Specifically, studies of modifiable protective factors are highly relevant.

The school constitutes a setting where children and young people spend a considerable amount of their time. It is also an arena which provides opportunities for modifiable protective factors, including access to supportive relations with teachers. Strong student–teacher relationships are central to so-called “effective schools”. Research into the scholarship of effective schools has shown that there are certain characteristics of schools that are linked with more favourable learning and behavioural outcomes among students, irrespective of the students’ own social background and the school’s student body composition [22]. Such features include high expectations of the students, well formulated and constructive feedback, strong relations between teachers and students as well as between the parents and the school, clear and transparent goals, and an orderly environment [22–24]. These features are reflected by the concept school ethos, referring to the norms, values, attitudes and behaviours permeating the social interaction patterns at a school [25]. In an article based on the same data that is used in the current study, we analysed the link between problematic familial alcohol use and heavy episodic drinking among senior-level students and investigated the buffering role of the school’s degree of teacher-rated student focus, i.e., one specific aspect of school ethos which reflects strong and positive teacher-student relationships [26]. Building upon these findings, a related study focused on the association between perceived problematic familial alcohol use and heavy episodic drinking among upper secondary students and examined the buffering role of several dimensions of teacher-rated school ethos [27]. Both these studies demonstrated an association between perceived problematic familial alcohol use and heavy episodic drinking among students, and that this relationship was weaker among students attending schools with higher levels of teacher-rated school ethos, suggesting a compensatory effect [26, 27].

Based on the literature on protective factors in children of parents with problematic alcohol use [15, 20, 21] which highlights the importance of social support from adults outside the closest family, it seems possible that a strong student focus at a school may be protective against certain stress-related complaints with origin in their family situation. This assumption is also in line with the stress-buffering model, which postulates that perceived or received social resources have a buffering role in the association between a stressor and stress-related outcomes [28].

Focusing on problematic drinking in the family as a stressor, the aim of the current study is to examine the association between perceived problematic familial alcohol use and students’ stress-related complaints, and specifically to explore if the school’s level of student focus can serve as a buffer in this relationship.

## Methods

### Data and participants

The data was drawn from four cross-sectional surveys performed in 2014 and in 2016 among ninth grade students and senior-level teachers in the same schools; the Stockholm School Survey (SSS) and the Stockholm Teacher Survey (STS), which were combined. Administrative register information on schools from the Swedish National Agency for Education has also been linked to the data.

The SSS is performed biennually by Stockholm Municipality among students in grade 9 of compulsory school (~ 15–16 years) and in grade 2 of upper secondary school (~ 17–18 years) in all public schools and in many of the independent schools in Stockholm. Students complete the questionnaires in the classroom with paper and pencil. The response rate for the 2014 and the 2016 surveys has been estimated to 78% [29] (p. 17, Table 2). The number of grade 9 students who participated was 5,245 in 2014 and 6,381 in 2016 [29] (p. 18).

The STS was conducted among teachers in 2014 and in 2016 as part of a research project at Stockholm University. Teachers in the same schools that participated in the SSS were invited to take part in a web survey about conditions in the school, with the purpose of aggregating this information to the school level and linking it to the student level data from the SSS. In 2014, the STS was performed among teachers in the senior-level schools that participated in the SSS, and in 2016 among teachers in both senior-level and upper secondary schools that took part in the SSS. School-level measures of teacher ratings of, e.g., school ethos, consensus and cooperation among teachers, and teachers’ time use, were formed by constructing indices and then calculating the mean value of each school. These measures were then linked to the student level data from the SSS. The response rate among senior-level teachers was 54% in both 2014 and in 2016 [29] (p. 17, Table 2). The number of senior-level teachers who participated was 1,286 in 2014 and 1,247 in 2016 [29] (p. 19).

The present study was based on data from the SSS collected among students in grade 9, the STS collected among senior-level teachers, and official register information on schools from the Swedish National Agency for Education. Data from 2014 and 2016 were pooled in order to increase the sample size. The number of ninth grade students which could be linked to teacher-level information was 10,757 [29] (p. 19). After exclusion of students with missing information on any of the study variables (n = 2,813), the study sample included responses from 7,944 students and 2,024 teachers distributed across 147 senior-level school units, i.e. 74% of the participating grade 9 students. More information on the data material is provided elsewhere [29].

### Ethics

The Regional Ethical Review Board of Stockholm has concluded that data from the Stockholm School Survey are not subject to consideration for ethical approval since the questionnaires are completed anonymously without any information on personal identification (ref. no. 2010/241–31/5). The Regional Ethical Review Board of Stockholm has provided ethical approval for the Stockholm Teacher Survey (ref. no. 2013/2188–31/5; 2015/1827–31/5). In accordance with the ethical permission, informed consent was obtained from those who participated. All methods were carried out in accordance with relevant guidelines and regulations. During the data processing for the current study, the data material was treated with caution and all analyses were performed and presented at the group level, protecting the confidentiality of the participants.

### Measures

Stress-related complaints were captured by two measures: co-occurring somatic complaints and psychological distress.

Co-occurring somatic complaints were constructed from two questions in the SSS about headache and stomach ache, respectively: “How often have you had headaches this school year?” and “How often this school year have you had an upset stomach (e.g., stomach ache, stomach cramps, upset stomach, nausea, wind, constipation or diarrhoea)?” The response categories were “Never”, “About once a term”, “About once a month”, “About once a week” and “Several times a week”. Students who marked that they had weekly co-occurring complaints, i.e., both headache and stomach ache about once a week or more often, were classified as having co-occurring somatic complaints. The co-occurrence of headache and stomach ache can be seen as a marker of stressful conditions [30]. The same measure has been used in prior studies [31, 32].

Psychological distress was based on three items in the SSS: “Do you feel sad and depressed without knowing why?”, “Do you ever feel frightened without knowing why?”, and “How often do you feel it is really good to be alive?”. The response categories were: “Seldom”, “Occasionally”, “Sometimes”, “Pretty often”, and “Very often”. Each item was coded 1–5 (the item on enjoyment of life was reversely coded). The three items had acceptable internal consistency (Cronbach’s α = 0.66). The values of each item were added to an index where higher values represented higher levels of psychological distress. The measure has been used previously [33].

Perceived problematic familial alcohol use was captured by one question in the SSS: “Do you think someone in your family drinks too much alcohol?” The response categories were “Yes”, “No”, and “Don’t know”. Students who replied “Don’t know” were coded as missing. The same question has been used in earlier studies [8, 26, 27].

Teacher-rated student focus was captured by four items in the STS: “At this school the teachers make an effort to provide positive feedback about students’ performance”, “Teachers have high expectations of student performance”, “Teachers at this school take their time with students even if they want to discuss something other than schoolwork”, and “At this school the students are treated with respect”. Response categories were on a five point-scale from “Strongly agree” to “Strongly disagree”. The items had high internal consistency (Cronbach’s α = 0.75). Values for all items were summed to an index with the possible range 4–20, with higher values indicating higher teacher ratings of the school’s student focus. In order to examine potentially non-linear associations, we constructed three categories of about equal size in order to distinguish students in schools with a relatively weak, intermediate, and strong student focus. The measure has been used in previous studies [26, 27, 34]. Student focus is a subdimension of the broader concept of school ethos. A confirmatory factor analysis of a measure of school ethos based on 12 items, including the four items on student focus, proved to have good model fit (CFI = 0.93; TLI = 0.92; RMSEA = 0.09) [25, 33].

A set of covariates that previously have been shown to be associated with both perceived problematic familial alcohol use and adolescent health [8] were also included to account for possible confounding. Accordingly, at the student level, we controlled for gender, family structure, parental university education, parental unemployment, and migration background, based on student reported information from the SSS. Furthermore, we included two control variables at the school level which may be correlated with the main variables of interest: the school’s segregation profile, with schools classified into “privileged”, “typical”, “deprived” and “deprived immigrant” school clusters based on Latent Class Analysis (LCA) (for a detailed description of the LCA and the measure, see [25, 29]); and the school’s student–teacher ratio (i.e., the number of students per teacher), based on official registry information from the Swedish National Agency for Education.

### Statistical method

To scrutinise the associations between perceived familial alcohol use and adolescent stress-related complaints by schools’ degree of student focus, we first carried out cross-tabulations with chi-square tests (for somatic complaints) and ANOVAs (for psychological distress). Next, we conducted two-level binary logistic regression models of somatic complaints using the *melogit* command in Stata, presenting odds ratios (OR) with 95% Confidence Intervals (CI), and two-level linear regression models of psychological distress using the *xtmixed* command, presenting unstandardised coefficients (b) with 95% Confidence Intervals (CI). For all models, the Intraclass Correlation (ICC) is reported, which indicates the amount of variation that can be attributed to the higher unit level, in this case the school level. All analyses were performed in Stata, version 17 [35].

## Results

Descriptive statistics of the study sample are presented in Table 1. In the data, 11.8% of the students reported co-occurring somatic complaints and 11.1% reported perceived problematic familial alcohol use. The study sample was evenly distributed by gender. About two thirds lived in two-parent households and one third in other family constellations. In the study sample, 59.2% had at least one parent with university education and 5.5% had at least one parent who was unemployed. With regards to migration background, 8.0% had lived in Sweden for less than ten years. The mean value of psychological distress was 6.38. At the school level, 18.1% of the students attended schools classified as “privileged”, 56.7% in “typical” schools, 11.6% in “deprived” schools and 13.7% in “deprived immigrant” schools. Teacher-rated student focus was classified into three categories of about equal size. The mean value of student–teacher ratio was 14.2, with a range between 8.7 and 23.7. Descriptive statistics of the full sample, including also cases with missing values, is provided in the Supplementary Information (Table S1). The distributions in Table 1 and in Table S1 do not indicate any substantial differences between the study sample and the full sample. Distributions of perceived parental alcohol use, co-occurring somatic complaints, and psychological distress by the student-level covariates are displayed in Table S2.

**Table 1:** Descriptives. *n* = 7944 students in 147 senior-level school units

	n	%		
*Student level*				
Co-occurring somatic complaints				
No	7006	88.2		
Yes	938	11.8		
Perceived problematic familial alcohol use				
No	7065	88.9		
Yes	879	11.1		
Gender				
Boy	3963	49.9		
Girl	3981	50.1		
Family structure				
Two-parent household	5303	66.8		
Other	2641	33.2		
Parental university education				
No or not known	3243	40.8		
At least one parent	4701	59.2		
Parental unemployment				
No parent unemployed	7508	94.5		
At least one parent unemployed	436	5.5		
Migration background				
≥ 10 years in Sweden	7312	92.0		
< 10 years in Sweden	532	8.0		
	Mean	s.d	Min	Max
Psychological distress	6.38	2.68	3	15
	n	%		
*School level*				
Teacher-rated student focus				
Weak	2649	33.3		
Intermediate	2722	34.3		
Strong	2573	32.4		
School segregation profile				
Privileged	1438	18.1		
Typical	4501	56.7		
Deprived	921	11.6		
Deprived immigrant	1084	13.7		
	Mean	s.d	Min	Max
Student–teacher ratio	14.2	2.6	8.7	23.7

Table 2 presents proportions of students reporting somatic complaints and mean levels of psychological distress by perceived problematic familial alcohol use, among all students and stratified by the school’s level of teacher-rated student focus. Chi-square tests and ANOVAs were performed to assess differences between groups.

**Table 2:** Proportions of students reporting co-occurring somatic complaints and mean values of psychological distress, by perceived problematic familial alcohol use, in the total sample and stratified by the school’s level of teacher-rated student focus. Differences between groups examined with χ^2^ tests and ANOVAs

	Total sample (*n* = 7944)		Weak teacher-rated student focus (*n* = 2649)		Intermediate teacher-rated student focus (*n* = 2722)		Strong teacher-rated student focus (*n* = 2573)	
Co-occurring somatic complaints								
Perceived problematic familial alcohol use	n	%	n	%	n	%	n	%
No	771	10.9	295	12.6	251	10.5	225	9.7
Yes	167	19.0	74	24.8	58	17.6	35	13.9
χ^2^		49.08***		32.91***		14.65***		4.51*
Psychological distress								
Perceived problematic familial alcohol use	Mean	s.d	Mean	s.d	Mean	s.d	Mean	s.d
No	6.23	0.03	6.30	0.06	6.24	0.05	6.14	0.05
Yes	7.58	0.09	8.00	0.17	7.28	0.15	7.47	0.17
ANOVA (F)		18.06***		9.81***		4.07***		5.73***

^***^p < 0.001 *p < 0.05

In the total study sample, co-occurring somatic complaints were reported by 10.9% of the students who had answered that there was no problematic familial alcohol use, whereas the share among students who had reported perceived problematic familial alcohol use was 19.0%. The difference was statistically significant. The analyses stratified by the school’s level of teacher-rated student focus showed that co-occurring somatic complaints were overall more prevalent among students who had reported perceived problematic familial alcohol use compared with those who had not, with statistically significant associations. However, the relative difference between the two categories varied by the level of the school’s student focus in a gradient manner. The relative difference was most substantive among students attending schools with a relatively weak student focus, where adolescents who perceived problematic familial alcohol use were almost twice as likely to report co-occurring somatic complaints (24.8/12.6 = 1.97) compared to students who did not report any such problems. Among students attending schools with an intermediate level of teacher-rated student focus, the elevated risk of co-occurring somatic complaints among those with perceived problematic familial alcohol use was somewhat lower (17.6/10.5 = 1.68), and among students attending schools with a strong student focus, the relative difference was even smaller (13.9/9.7 = 1.43).

In the total study sample, the mean value of psychological distress was 6.23 among the students who did not report perceived problematic familial alcohol use, and by 7.58 among those who did. The difference was statistically significant. The relative difference in psychological distress between those who did and those who did not report perceived problematic familial alcohol use varied by the level of the school’s degree of student focus, albeit not in a gradient manner. The relative difference in mean values of psychological distress was largest in schools with a relatively weak student focus (8.00/6.30 = 1.27). The relative difference was smallest among students in schools with an intermediate degree of student focus (7.28/6.24 = 1.17), followed by students in schools with a strong degree of student focus (7.47/6.14 = 1.22).

To further examine the associations between perceived problematic familial alcohol use and stress-related complaints, we performed a series of two-level binary logistic regression analyses of co-occurring somatic complaints (with results presented in Table 3) and a series of two-level linear regression analyses of psychological distress (with results presented in Table 4).

**Table 3:** Odds ratios (OR) and 95% confidence intervals (95% CI) from two-level binary logistic regressions of co-occurring somatic complaints. n = 7944 students in 147 senior-level school units. All models are adjusted for study year

	Model 1		Model 2		Model 3		Model 4	
	OR	95% CI	OR	95% CI	OR	95% CI	OR	95% CI
*Student level*								
Perceived problematic familial alcohol use								
No (ref.)	1.00	-	1.00	-	1.00	-	1.00	-
Yes	1.74***	1.43, 2.11	1.73***	1.43, 2.11	1.75***	1.44, 2.13	1.75***	1.44, 2.12
Gender								
Boy (ref.)	1.00	-	1.00	-	1.00	-	1.00	-
Girl	3.17***	2.71, 3.70	3.18***	2.72, 3.71	3.16***	2.70, 3.69	3.16***	2.71, 3.69
Family structure								
Two-parent household (ref.)	1.00	-	1.00	-	1.00	-	1.00	-
Other	1.19*	1.02, 1.38	1.18*	1.02, 1.37	1.18*	1.01, 1.37	1.18*	1.01, 1.37
Parental university education								
No or not known (ref.)	1.00	-	1.00	-	1.00	-	1.00	-
At least one parent	0.91	0.79, 1.06	0.94	0.81, 1.08	0.96	0.83, 1.12	0.96	0.83, 1.12
Parental unemployment								
No parent unemployed (ref.)	1.00	-	1.00	-	1.00	-	1.00	-
At least one parent unemployed	1.57**	1.21, 2.05	1.56**	1.20, 2.03	1.55**	1.19, 2.01	1.55**	1.19, 2.01
Migration background								
≥ 10 years in Sweden (ref.)	1.00	-	1.00	-	1.00	-	1.00	-
< 10 years in Sweden	1.04	0.80, 1.34	1.01	0.78, 1.30	0.97	0.74, 1.26	0.96	0.74, 1.25
*School level*								
Teacher-rated student focus								
Weak (ref.)			1.00	-			1.00	-
Intermediate			0.81*	0.66, 0.98			0.85	0.69, 1.04
Strong			0.70**	0.57, 0.86			0.76*	0.61, 0.97
School segregation profile								
Privileged (ref.)					1.00	-	1.00	-
Typical					1.13	0.88, 1.45	1.02	0.79, 1.33
Deprived					1.46*	1.06, 2.01	1.25	0.88, 1.76
Deprived immigrant					1.28	0.89, 1.83	1.11	0.76, 1.62
Student–teacher ratio					0.98	0.93, 1.02	0.98	0.94, 1.02
Intraclass Correlation (ICC)	2.5%		1.9%		2.0%		1.7%	
Perceived problematic familial alcohol use*							*0.80*	*0.51, 1.24*
*Intermediate teacher-rated student focus*							*(p* = *0.317)*	
Perceived problematic familial alcohol use*							*0.61*	*0.37, 1.00*
*Strong teacher-rated student focus*							*(p* = *0.051)*	

Model 1: Student level variables; Model 2: Student level variables + teacher-rated student focus; Model 3: Student-level variables + school segregation profile + student–teacher ratio; Model 4: Student level variables + teacher-rated student focus + school segregation profile + student–teacher ratio

^***^p < 0.001 **p < 0.01 *p < 0.05

**Table 4:** Unstandardised coefficients (b) and 95% confidence intervals (95% CI) from two-level linear regressions of psychological distress. *n* = 7944 students in 147 senior-level school units. All models are adjusted for study year

	Model 1		Model 2		Model 3		Model 4	
	b	95% CI	b	95% CI	b	95% CI	b	95% CI
*Student level*								
Perceived problematic familial alcohol use								
No (ref.)	0.00	-	0.00	-	0.00	-	0.00	-
Yes	1.12***	0.94, 1.29	1.12***	0.94, 1.29	1.12***	0.94, 1.29	1.12***	0.94, 1.29
Gender								
Boy (ref.)	0.00	-	0.00	-	0.00	-	0.00	-
Girl	1.80***	1.69, 1.91	1.80***	1.70, 1.91	1.80***	1.70, 1.91	1.80***	1.70, 1.91
Family structure								
Two-parent household (ref.)	0.00	-	0.00	-	0.00	-	0.00	-
Other	0.33***	0.21, 0.45	0.33***	0.21, 0.45	0.32***	0.20, 0.44	0.32***	0.20, 0.44
Parental university education								
No or not known (ref.)	0.00	-	0.00	-	0.00	-	0.00	-
At least one parent	-0.19**	-0.30, -0.08	-0.18**	-0.30, -0.07	-0.15*	-0.27, -0.03	-0.15*	-0.27, -0.03
Parental unemployment								
No parent unemployed (ref.)	0.00	-	0.00	-	0.00	-	0.00	-
At least one parent unemployed	0.43***	0.19, 0.67	0.43**	0.19, 0.67	0.42**	0.18, 0.66	0.42**	0.18, 0.66
Migration background								
≥ 10 years in Sweden (ref.)	0.00	-	0.00	-	0.00	-	0.00	-
< 10 years in Sweden	0.49***	0.28, 0.69	0.48***	0.27, 0.68	0.45***	0.24, 0.66	0.45***	0.24, 0.66
*School level*								
Teacher-rated student focus								
Weak (ref.)			0.00	-			0.00	-
Intermediate			-0.04	-0.20, 0.12			-0.00	-0.16, 0.16
Strong			-0.13	-0.29, 0.04			-0.01	-0.19, 0.17
School segregation profile								
Privileged (ref.)					0.00	-	0.00	-
Typical					0.23*	0.05, 0.42	0.23*	0.03, 0.43
Deprived					0.36**	0.10, 0.61	0.35*	0.07, 0.62
Deprived immigrant					0.33*	0.05, 0.61	0.33*	0.03, 0.62
Student–teacher ratio					0.00	-0.03, 0.03	0.00	-0.03, 0.03
Intraclass Correlation (ICC)	0.8%		0.7%		0.6%		0.6%	
Perceived problematic familial alcohol use* *Intermediate teacher-rated student focus*							-0.62** *(p* = *0.003)*	*-1.03, -0.20*
Perceived problematic familial alcohol use* *Strong teacher-rated student focus*							-0.48* *(p* = *0.033)*	*-0.92, -0.04*

Model 1: Student level variables; Model 2: Student level variables + teacher-rated student focus; Model 3: Student-level variables + school segregation profile + student–teacher ratio; Model 4: Student level variables + teacher-rated student focus + school segregation profile + student–teacher ratio

^***^p < 0.001 **p < 0.01 *p < 0.05

The first model in Table 3, including only student-level variables, shows that perceived problematic familial alcohol use was clearly associated with an increased likelihood of reporting co-occurring complaints (OR 1.74, 95% CI 1.43, 2.11). Furthermore, the model shows that girls were more likely than boys to report such complaints (OR 3.17, 95% CI 2.71, 3.70). Students who did not live in two-parent households also had a higher likelihood of reporting co-occurring somatic complaints (OR 1.19, 95% CI 1.02, 1.38). There was no statistically significant association between having university-educated parents and co-occurring somatic complaints (OR 0.91, 95% CI 0.79, 1.06). However, students with at least one unemployed parent were more likely to report co-occurring somatic complaints (OR 1.57, 95% CI 1.21, 2.05). Finally, migration background was not associated with the likelihood of reporting co-occurring somatic complaints (OR 1.04, 95% CI 0.80, 1.34). Model 2 added a school-level measure of teacher-rated student focus at the respective schools, which showed an inverse, graded association with co-occurring somatic complaints, (intermediate student focus: OR 0.81, 95% CI 0.66, 0.98, p = 0.034; strong student focus: OR 0.70, 95% CI 0.57, 0.86, p = 0.001). In Model 3, the segregation profile and student–teacher ratio of the school were included along with the full set of student-level variables. Vis-à-vis students in “privileged” schools, those in “typical” and in “deprived immigrant” schools did not differ in their reports of co-occurring somatic complaints. However, students attending “deprived” schools had a higher likelihood of reporting co-occurring somatic complaints compared with students in “privileged” schools. No statistically significant association was seen for the school’s student–teacher ratio and co-occurring somatic complaints at the student level. Model 4 included all student- and school-level variables. The association between teacher-rated student focus and students’ likelihood of reporting co-occurring somatic remained statistically significant in this model. Finally, we tested for the cross-level interaction between teacher-rated student focus and perceived problematic familial alcohol use to the fully adjusted model. The estimate for strong student focus was negative and very close to statistically significant (OR 0.61, 95% CI 0.37, 1.00, *p* = 0.051), indicating that the association between perceived parental alcohol problems on psychological distress was weaker in schools with a strong level of student focus. Analyses of co-occurring somatic complaints stratified by gender are displayed in the Supplementary Material, Table S3 (boys) and Table S4 (girls). The results show that the cross-level interaction was driven by boys.

Table 4 displays results from the analyses of psychological distress. Model 1 shows that perceived problematic familial alcohol use was clearly associated with higher levels of psychological distress (b = 1.12, 95% CI 0.94, 1.29). Psychological distress was higher in girls than boys (b = 1.80, 95% CI 1.69, 1.91). Higher levels of psychological distress were observed among students who did not live in two-parent households (b = 0.33, 95% CI 0.21, 0.45), who had at least one unemployed parent (b = 0.43, 95% CI 0.19, 0.67), and who had lived less than 10 years in Sweden (b = 0.49, 95% CI 0.28, 0.69). Conversely, having a parent with university education was associated with lower levels of psychological distress (b = -0.19, 95% CI -0.30, -0.08). Model 2 added teacher-rated student focus, which did not show any statistically significant association with students’ psychological distress. In Model 3, the segregation profile and student–teacher ratio of the school were included along with the student level variables. Compared with students in “privileged” schools, the level of psychological distress was higher among students in “typical” (b = 0.23, 95% CI 0.05, 0.42), in “deprived” (b = 0.36, 95% CI 0.10, 0.61), and in “deprived immigrant” schools (b = 0.33, 95% CI 0.05, 0.61). In Model 4, mutually adjusting for the full set of student and school level variables, all associations remained similar to the previous models. Finally, we added a cross-level interaction between teacher-rated student focus and perceived problematic familial alcohol use to the fully adjusted model. The estimates were negative and statistically significant, indicating that the association between perceived parental alcohol problems and psychological distress was weaker in schools with an intermediate (b = -0.62, 95% CI -1.03, -0.20, p = 0.003) and a strong level of student focus (b = -0.48, 95% CI -0.92, -0.04, p = 0.033), compared with schools with a weak student focus. Analyses of psychological distress stratified by gender are displayed in the Supplementary Material, Table S5 (boys) and Table S6 (girls). Similar to the analyses of co-occurring somatic complaints, these results also show that the cross-level interaction was largely driven by boys.

**Fig. 1:**
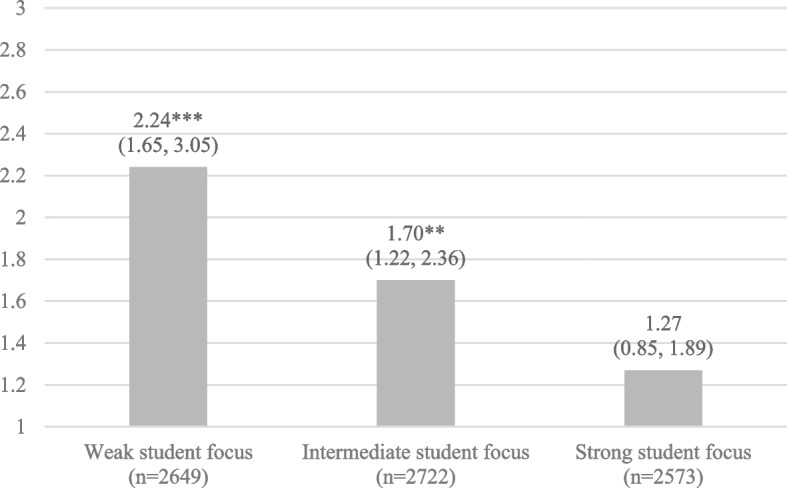
Odds ratios (OR) and 95% confidence intervals (95% CI) displaying the association between perceived problematic familial alcohol use and adolescents’ co-occurring somatic complaints, stratified by the school’s level of teacher-rated student focus (reference category = no perceived problematic familial alcohol use)

To further illustrate the difference in the association between perceived problematic familial alcohol use and co-occurring somatic complaints among students in schools with different levels of teacher-rated student focus, we performed three fully adjusted multilevel models stratified by the school’s level of student focus (with the sample split into the same three categories as in the stratified analyses reported in Table 2). The results, presented in Fig. 1, show that the association between perceived problematic familial alcohol use and co-occurring somatic complaints was strongest for students in schools with a relatively weak student focus (OR 2.24, 95% CI 1.65, 3.05, *p* < 0.001), somewhat less pronounced for those attending schools with an intermediate student focus (OR 1.70, 95% CI 1.22, 2.36, *p* < 0.01), and even weaker and non-significant for students attending schools with a relatively strong student focus (OR 1.27, 95% CI 0.85, 1.89, *p* = 0.244).

**Fig. 2:**
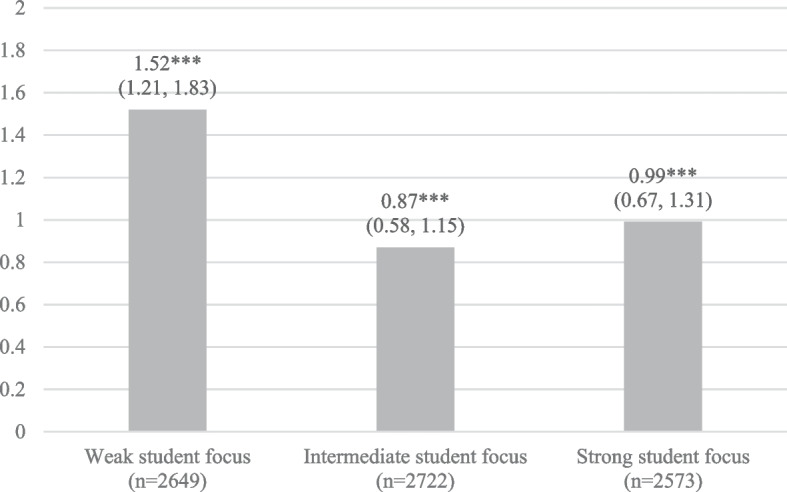
Unstandardised coefficients (b) and 95% confidence intervals (95% CI) displaying the association between perceived problematic familial alcohol use and adolescents’ psychological distress, stratified by the school’s level of teacher-rated student focus (reference category = no perceived problematic familial alcohol use)

Finally, we also performed three sets of fully adjusted multilevel analyses of the association between perceived problematic familial alcohol use and psychological distress, stratified by the school’s level of student focus. The results are displayed in Fig. 2. The graphs show that the coefficient of perceived problematic familial alcohol use was largest in the analyses of students in schools with a relatively weak student focus (b = 1.52, 95% CI 1.21, 1.83, *p* < 0.001), and smaller in the analyses of students in schools with an intermediate (b = 0.87, 95% CI 0.58, 1.15, *p* < 0.001) and a strong student focus (b = 0.99, 95% CI 0.67, 1.31, *p* < 0.001).

## Discussion

This study examined the relationship between perceived problematic familial alcohol use and adolescents’ stress-related complaints, and the buffering role of the school’s student focus in this association.

The results showed that perceived problematic familial alcohol use was clearly linked with a higher likelihood of reporting co-occurring somatic complaints and reporting higher levels of psychological distress among students. These findings reflect those from earlier studies which have shown problematic drinking among parents to be associated with a range of adverse mental health outcomes [3–13]. Overall, these studies indicate that perceived problematic familial alcohol use is a severe stressor. Possible mechanisms in the association between perceived problematic familial alcohol use and stress-related complaints include, e.g., feelings of fear, stigma, shame, and guilt [14–16], neglectful, inconsistent and unpredictable parenting [15, 16], poor parent–child relationships [3, 18, 20], and parentification, i.e., a reversal of the responsibility roles in the family [15, 16, 36, 37]. Another possible mechanism is that adolescents with perceived problematic familial alcohol use are more inclined to drink alcohol themselves [38], partly as a way to cope with the stress [16]. Excessive drinking is, in turn, associated with poorer mental health [39]. Additionally, living with a parent with problematic alcohol use may include difficulties with friendships, social isolation, and development of peer relationships which may include antisocial activities and risky behaviours [15]. It should however be stressed that parental problematic alcohol use often co-occurs with other adverse conditions in the family [17–19]. For instance, children exposed to parental problematic alcohol use are also more likely to experience or witness violence, neglect, and abuse [15]. In the current study, we did not adjust for other adverse childhood experiences, and hence we cannot exclude the possibility that there are other aspects of household dysfunction which account for the association between problematic familial alcohol use and adolescents’ stress-related complaints.

The school’s degree of teacher-rated student focus was negatively associated with co-occurring somatic complaints, but not with psychological distress when the school’s segregation profile was adjusted for. Furthermore, our analyses showed a buffering effect of teachers’ ratings of the school’s student focus on the association between perceived problematic familial alcohol use and co-occurring somatic complaints: A cross-level interaction revealed that the association between perceived problematic familial alcohol use and co-occurring somatic complaints was weaker in schools with a strong student focus. Regarding psychological distress, the results showed a buffering effect of both intermediate and strong student focus. These results are in line with prior studies based on the same data as the current one, which showed that the school’s degree of student focus served as a buffer in the association between perceived problematic alcohol use in the family and students’ own alcohol use [26, 27]. The findings also align with previous research which emphasises social support resources outside the family as important protective factors for children of parents with problematic alcohol use [15, 20, 21].

The moderating effect of the school’s degree of student focus on the association between perceived problematic familial alcohol used and adolescent stress-related complaints may be interpreted in light of the scholarship of effective schools. This field of research presents that certain characteristics of schools such as high expectations, clear goals, strong teacher-student and parent-school relations, and an orderly environment, can induce more favourable outcomes in their students in terms of higher academic achievement [22, 25], but also fewer behavioural problems [23, 34] and less psychological distress [33]. More specifically, the school’s level of student focus, which was at the heart of the current study, captures the degree of strong and positive relations between teachers and students at a school. An interpretation of the buffering role of student focus in the association between problematic familial drinking and co-occurring somatic complaints among students is that teachers act as important adults who, through their presence and support, contribute to making the situation of students with problematic conditions in the home more manageable, thereby inducing resilience. In a systematic review of qualitative studies of children of substance users, Muir et al. [16] reported that the school was often brought up as a safe and supportive place, but was also associated with risks. Young people acknowledged the importance of achieving well in school, but due to their home situation they often felt worried, had to struggle with homework, and sometimes also had to face consequences for unacceptable behaviour, which in turn led to a reduced access to social and professional support. As highlighted by Muir et al. [16]: “Young people reported wanting school staff to recognize the impacts of parental substance use on children, to improve referral and early access to support” (p. 11). One interpretation of our findings is that teachers in schools characterised by a strong student focus have better possibilities to recognise and support students with problematic situations in the home.

Additional analyses in the current study showed that the moderating effect of teacher-rated student focus was driven by boys. This result aligns with findings from previous studies which showed that girls were less affected by parental problematic alcohol use in the short term, and that individual-level characteristics were more important for the resilience of females but that external support was more important for males [15].

However, it should be acknowledged that the total amount of variation in stress-related complaints that could be attributed to the school level was minor, as indicated by the ICCs. In other words, most of the variation in stress-related complaints is due to factors at the individual student level. Hence, in order to tackle stress-related complaints in more general terms, improving the school’s degree of student focus is not sufficient.

The statistically significant associations between several of the covariates and stress-related complaints deserve to be mentioned. Girls were more likely to report co-occurring somatic complaints and psychological distress, reflecting prior research (e.g., [30, 40]). In addition, not living with two original parents and parental unemployment were linked with a higher likelihood of both studied outcomes. Students with no university-educated parent and those who had lived in Sweden less than 10 years were more likely to report psychological distress, whereas no statistically significant differences were seen for co-occurring somatic complaints. In all, the findings point at clear social inequalities in stress-related complaints. Although this was not the focus of the current study, scrutinising potential explanations behind such inequalities is a relevant task for future research.

The main strength of the study is that the data material was based on information collected among both teachers and students, as well as linked register information, thus reducing the risk of common methods variance. In particular, it is a benefit that the schools’ degree of student-focus and stress-related complaints were assessed by teachers and students, respectively, since we thereby minimise the possibility that negative affectivity influences the results [41]. Nonetheless, there are also limitations. It should be acknowledged that even though the measures have been used in previous studies, they have not all been formally validated. With regards to the measure of perceived problematic familial drinking, it should also be emphasised that this is subjective from the students’ point of view. The variable may reflect adolescents’ awareness of familial drinking problems (rather than the actual occurrence) but also their inclination to disclose information about sensitive experiences (for a discussion, see [8]). Additionally, the measure is rather crude since it is based on only one question, and it does not specify which persons in the family that have problematic alcohol use. Another limitation that should be acknowledged is the non-response. With regards to external non-response, it is possible students with problematic situations in the home and/or who suffer from mental health problems were less likely to be at school at the day of the survey, and may hence be underrepresented in the data. There was also a relatively high degree of internal non-response in the survey, i.e., students who participated but who skipped certain questions. A comparison of the distribution of the variables in the full sample vs. our study sample did however not show any substantial differences. Further, the cross-sectional nature of the data limits possibilities of causal interpretations. Relatedly, although we controlled for a range of sociodemographic characteristics at the student level and for school segregation profile and student–teacher ratio at the school level, it is possible that we were not able to fully account for the selection of students with certain features into certain schools, meaning that there may be unmeasured characteristics that could affect the associations. Finally, it should be mentioned that the study was conducted among senior-level schools in Stockholm, Sweden. To be able to generalise the findings, studies of other age groups and other national or educational settings are needed to corroborate the results. However, the fact that the study was conducted in this setting also makes a contribution to the research field. The systematic review by Wlodarczyk et al. [21] included 11 studies of protective mental health factors in children and parents with substance use disorders, of which 10 were conducted in the US and one in Israel. Accordingly, studies conducted in other contexts are relevant. Future studies could also benefit from considering other aspects of the school context as well as other types of family stressors when examining the potentially buffering role of the school for students’ stress-related outcomes.

## Conclusion

The results of the current study provide support for the assumption that schools with an intermediate or strong student focus can buffer against young people’s exposure to problematic conditions in the family, thereby serving a compensatory role. These results, taken together with our previous studies, indicate that strengthening schools’ ability to operate in accordance with the principles of effective schools may benefit students who experience stressors in their family life. Specifically, providing teachers with sufficient time and resources for creating and maintaining strong relations with their students is a task that should be given adequate attention from policy-makers.

### Supplementary Information

**Additional file 1.** 

## Data Availability

The data that support the findings of this study are available from Stockholm Municipality (the Stockholm School Survey) and the Department of Public Health Sciences, Stockholm University (the Stockholm Teacher Survey) but restrictions apply to the availability of these data, which were used under license for the current study, and so are not publicly available. Data are however available from the corresponding authors upon reasonable request (contact Professor Bitte Modin: bitte.modin@su.se) and with permission of Stockholm Municipality (e-mail: stockholmsenkaten@stockholm.se) and the Department of Public Health Sciences, Stockholm University.

## Abbreviations

95% CI: 95% Confidence Interval
ICC: Intraclass Correlation
OR: Odds Ratio
SSS: Stockholm School Survey
STS: Stockholm Teacher Survey

## Footnotes

1 An earlier (work in progress) version of the study was presented at the European Public Health Conference in 2021, and the conference abstract was accordingly published in the European Journal of Public Health [1]. In the first version that was presented, only one outcome was analysed (co-occurring somatic complaints), whereas the full manuscript includes analyses of both co-occurring somatic complaints and psychological distress.

## References

[1] Brolin Låftman S, Magnusson C, Olsson G, Svensson J, Wahlström J, Modin B. Problematic alcohol use in the family and adolescents’ stress-related complaints. Eur J Public Health. 2021; 31(Supplement_3). 10.1186/s12889-023-16505-x PMC10492349 37684584

[2] Elgán TH, Berman AH, Jayaram-Lindström N, Hammarberg A, Jalling C, Källmén H (2021). Psychometric properties of the short version of the children of alcoholics screening test (CAST-6) among Swedish adolescents. Nord J Psychiatry.

[3] Pisinger VS, Bloomfield K, Tolstrup JS (2016). Perceived parental alcohol problems, internalizing problems and impaired parent—child relationships among 71 988 young people in Denmark. Addiction.

[4] Pisinger VSC, Tolstrup JS. Are emotional symptoms and depression among young people with parental alcohol problems modified by socioeconomic position? Eur Child Adolesc Psychiatry. 2022;31:747–55. 10.1007/s00787-020-01716-z 33432403

[5] Ramstedt M, Raninen J, Larm P, Livingston M (2022). Children with problem drinking parents in Sweden: Prevalence and risk of adverse consequences in a national cohort born in 2001. Drug Alcohol Rev.

[6] Ramstedt M, Raninen J, Larm P, Livingston M (2023). Children with problem-drinking parents in a Swedish national sample: is the risk of harm related to the severity of parental problem drinking?. Eur J Pub Health.

[7] Wahlström J, Magnusson C, Låftman SB, Svensson J (2023). Parents’ drinking, childhood hangover? Parental alcohol use, subjective health complaints and perceived stress among Swedish adolescents aged 10–18 years. BMC Public Health.

[8] Wahlström J, Magnusson C, Svensson J, Låftman SB: Problematic familial alcohol use and adolescent outcomes: Do associations differ by parental education? Nordic Stud Alcohol Drugs. 2023:14550725231157152. 10.1177/14550725231157152 PMC10688401 38045008

[9] Syed NR, Wahlström J, Låftman SB, Svensson J (2023). Perceived parental alcohol problems and psychosomatic complaints among adolescents in Sweden. Addict Behav Rep.

[10] Raitasalo K, Holmila M, Jääskeläinen M, Santalahti P (2019). The effect of the severity of parental alcohol abuse on mental and behavioural disorders in children. Eur Child Adolesc Psychiatry.

[11] Rossow I, Moan IS (2012). Parental intoxication and adolescent suicidal behavior. Arch Suicide Res.

[12] Pisinger VS, Hawton K, Tolstrup JS (2018). Self-injury and suicide behavior among young people with perceived parental alcohol problems in Denmark: a school-based survey. Eur Child Adolesc Psychiatry.

[13] Landberg J, Danielsson AK, Hemmingsson T (2019). Fathers’ alcohol use and suicidal behaviour in offspring during youth and young adulthood. Acta Psychiatr Scand.

[14] Tamutienė I, Jogaitė B (2019). Disclosure of alcohol-related harm: Children’s experiences. Nordic Stud Alcohol Drugs.

[15] Velleman R, Templeton L (2007). Understanding and modifying the impact of parents' substance misuse on children. Adv Psychiatr Treat.

[16] Muir C, Adams EA, Evans V, Geijer-Simpson E, Kaner E, Phillips SM, Salonen D, Smart D, Winstone L, McGovern R. A systematic review of qualitative studies exploring lived experiences, perceived impact, and coping strategies of children and young people whose parents use substances. Trauma Violence Abuse. 2022:15248380221134297. 10.1177/15248380221134297 PMC10594843 36384375

[17] Felitti VJ, Anda RF, Nordenberg D, Williamson DF, Spitz AM, Edwards V, Marks JS (1998). Relationship of childhood abuse and household dysfunction to many of the leading causes of death in adults: The Adverse Childhood Experiences (ACE) Study. Am J Prev Med.

[18] Dube SR, Anda RF, Felitti VJ, Croft JB, Edwards VJ, Giles WH (2001). Growing up with parental alcohol abuse: exposure to childhood abuse, neglect, and household dysfunction. Child Abuse Negl.

[19] Petruccelli K, Davis J, Berman T (2019). Adverse childhood experiences and associated health outcomes: a systematic review and meta-analysis. Child Abuse Negl.

[20] Park S, Schepp KG (2015). A systematic review of research on children of alcoholics: Their inherent resilience and vulnerability. J Child Fam Stud.

[21] Wlodarczyk O, Schwarze M, Rumpf H-J, Metzner F, Pawils S (2017). Protective mental health factors in children of parents with alcohol and drug use disorders: a systematic review. PLoS One.

[22] Rutter M. Fifteen thousand hours: Secondary schools and their effects on children. Cambridge: Harvard University Press; 1979.

[23] West P, Sweeting H, Leyland A. School effects on pupils' health behaviours: evidence in support of the health promoting school. Res Papers Educ. 2004, 19(3):261–291.

[24] Wang AH, Walters AM, Thum Y (2013). Identifying highly effective urban schools: Comparing two measures of school success. Int J Educ Manag.

[25] GranvikSaminathen M, Brolin Låftman S, Almquist YB, Modin B (2018). Effective schools, school segregation, and the link with school achievement. Sch Eff Sch Improv.

[26] Olsson G, Brolin Låftman S, Modin B (2019). Problematic familial alcohol use and adolescents’ heavy drinking: can conditions in school compensate for the increased risk of heavy drinking among adolescents from families with problematic alcohol use?. Int J Adolesc Youth.

[27] Olsson G, Brolin Låftman S, Wahlström J, Modin B (2021). Problematic familial alcohol use and heavy episodic drinking among upper secondary students: a moderator analysis of teacher-rated school ethos. BMC Res Notes.

[28] Cohen S, Wills TA (1985). Stress, social support, and the buffering hypothesis. Psychol Bull.

[29] Kjellström J, Holmin von Saenger I, Jarl EL. Modin B: Technical report for the Teacher Survey with linkage to the Stockholm School Survey. 2018.

[30] Alfven G, Östberg V, Hjern A (2008). Stressor, perceived stress and recurrent pain in Swedish schoolchildren. J Psychosom Res.

[31] Modin B, Karvonen S, Rahkonen O, Östberg V (2015). School performance, school segregation, and stress-related symptoms: Comparing Helsinki and Stockholm. Sch Eff Sch Improv.

[32] Alm S, Låftman SB (2018). The gendered mirror on the wall: Satisfaction with physical appearance and its relationship to global self-esteem and psychosomatic complaints among adolescent boys and girls. Young.

[33] GranvikSaminathen M, Plenty S, Modin B (2021). The role of academic achievement in the relationship between school ethos and adolescent distress and aggression: a study of ninth grade students in the segregated school landscape of Stockholm. J Youth Adolesc.

[34] Modin B, Låftman SB, Östberg V (2017). Teacher rated school ethos and student reported bullying—a multilevel study of upper secondary schools in Stockholm, Sweden. Int J Environ Res Public Health.

[35] StataCorp: Stata Statistical Software: Release 17. In. College Station, TX: StataCorp LLC; 2021.

[36] Hedges KE (2012). A family affair: contextual accounts from addicted youth growing up in substance using families. J Youth Stud.

[37] Bender AK, Bucholz KK, Edenberg HJ, Kramer JR, Anokhin AP, Meyers JL, Kuperman S, Hesselbrock V, Hesselbrock M, McCutcheon VV (2020). Trauma exposure and post-traumatic stress disorder among youth in a high-risk family study: associations with maternal and paternal alcohol use disorder. J Family Trauma Child Custody Child Dev.

[38] Rossow I, Keating P, Felix L, McCambridge J (2016). Does parental drinking influence children's drinking? A systematic review of prospective cohort studies. Addiction.

[39] Griswold MG, Fullman N, Hawley C, Arian N, Zimsen SR, Tymeson HD, Venkateswaran V, Tapp AD, Forouzanfar MH, Salama JS (2018). Alcohol use and burden for 195 countries and territories, 1990–2016: a systematic analysis for the Global Burden of Disease Study 2016. Lancet.

[40] Wiklund M, Malmgren-Olsson E-B, Öhman A, Bergström E, Fjellman-Wiklund A (2012). Subjective health complaints in older adolescents are related to perceived stress, anxiety and gender–a cross-sectional school study in Northern Sweden. BMC Public Health.

[41] Watson D, Clark LA. Negative affectivity: the disposition to experience aversive emotional states. Psychol Bull. 1984;96(3):465–90. 6393179

