# Supplementary material for: Perceived problematic alcohol use in the family and adolescents’ stress-related complaints: examining the buffering role of the school’s degree of student focus as rated by its teachers
Source: BMC Public Health. 2023 Sep 9;23:1754. doi: 10.1186/s12889-023-16505-x (PMC10492349; doi:10.1186/s12889-023-16505-x)
Supplement: Supplementary file 1 — Additional file 1. [file 12889_2023_16505_MOESM1_ESM.pdf]

Supplementary Information. Table S1. Descriptives of the full study sample (n=10,757 students in 169 school units)

	n	%	
Student level			
Co-occurring somatic complaints			
No	8688	87.6	
Yes	1236	12.4	
Missing	833	-	
Perceived problematic familial alcohol use			
No	8658	89.0	
Yes	1076	11.1	
Missing	1023	-	
Gender			
Boy	5179	50.2	
Girl	5139	49.8	
Missing	439	-	
Family structure			
Two-parent household	6976	64.9	
Other	3781	35.2	
Missing	0		
Parental university education			
No or not known	4569	42.5	
At least one parent	6188	57.5	
Missing	0	-	
Parental unemployment			
No parent unemployed	10103	93.9	
At least one parent unemployed	654	6.1	
Missing	0	-	
Migration background			
≥10 years in Sweden	9539	89.9	
<10 years in Sweden	1073	10.1	
Missing	145	-	
	n	Mean	s.d.
Psychological distress	9749	6.47	2.73
Missing	1008	-	-
	n	%	
School level			
Teacher-rated student focus (N=169 school units)			
Weak	3637	33.8	
Intermediate	3652	34.0	
Strong	3468	32.2	
Missing	0	-	
School segregation profile (N=150 school units)			
Privileged	1856	18.0	
Typical	5785	56.2	
Deprived	1168	11.4	
Deprived immigrant	1479	14.4	
Missing	469	-	
	n	Mean	s.d.
Student-teacher ratio (N=162 school units)	10404	14.0	2.7
Missing	353	-	-

Supplementary Information. Table S2. Perceived problematic familial alcohol use, co-occurring somatic complaints, and psychological distress by student-level covariates. Differences between groups examined with χ^2 tests and ANOVAs. (n=10,757 students in 169 school units)

	Perceived problematic familial alcohol use		Co-occurring somatic complaints		Psychological distress	
	%	χ^2	%	χ^2	Mean	ANOVA (F)
Gender						
Boy	9.5		6.1		5.45	
Girl	12.6	18.73***	17.5	244.65***	7.30	99.16***
Family structure						
Two-parent household	8.1		10.8		6.19	
Other	17.1	145.31***	13.8	15.39***	6.74	6.87***
Parental university education						
No or not known	12.3		12.5		6.51	
At least one parent	10.2	8.54**	11.4	2.22	6.28	2.02*
Parental unemployment						
No parent unemployed	10.8		11.4		6.34	
At least one parent unemployed	16.3	12.77***	19.0	23.15***	7.07	3.87***
Migration background						
≥10 years in Sweden	11.1		11.7		6.33	
<10 years in Sweden	10.3	0.42	13.0	0.90	6.92	4.86***

***p<0.001 **p<0.01 *p<0.05

Supplementary Information. Table S3. Odds ratios (OR) and 95% confidence intervals (95% CI) from two-level binary logistic regressions of co-occurring somatic complaints among boys. n=3963 students in 147 senior-level school units. All models are adjusted for study year.

	Model 1		Model 2		Model 3		Model 4	
	OR	95% CI	OR	95% CI	OR	95% CI	OR	95% CI
Student level								
Perceived problematic familial alcohol use								
No (ref.)	1.00	-	1.00	-	1.00	-	1.00	-
Yes	2.57***	1.83, 3.61	2.60***	1.85, 3.66	2.57***	1.83, 3.62	2.60***	1.85, 3.67
Family structure								
Two-parent household (ref.)	1.00	-	1.00	-	1.00	-	1.00	-
Other	1.00	0.75, 1.33	1.00	0.76, 1.33	0.99	0.75, 1.32	1.00	0.75, 1.33
Parental university education								
No or not known (ref.)	1.00	-	1.00	-	1.00	-	1.00	-
At least one parent	0.75*	0.57, 0.98	0.78	0.59, 1.02	0.79	0.60, 1.04	0.80	0.61, 1.06
Parental unemployment								
No parent unemployed (ref.)	1.00	-	1.00	-	1.00	-	1.00	-
At least one parent unemployed	1.45	0.86, 2.42	1.40	0.83, 2.35	1.42	0.85, 2.38	1.39	0.83, 2.33
Migration background								
≥10 years in Sweden (ref.)	1.00	-	1.00	-	1.00	-	1.00	-
<10 years in Sweden	1.53*	1.02, 2.31	1.48	0.99, 2.24	1.45	0.95, 2.23	1.46	0.95, 2.23
School level								
Teacher-rated student focus								
Weak (ref.)			1.00	-			1.00	-
Intermediate			0.66*	0.48, 0.91			0.68*	0.49, 0.94
Strong			0.78	0.57, 1.08			0.85	0.59, 1.22
School segregation profile								
Privileged (ref.)					1.00	-	1.00	-
Typical					1.13	0.75, 1.68	1.12	0.73, 1.72
Deprived					1.51	0.90, 2.53	1.42	0.80, 2.51
Deprived immigrant					1.39	0.76, 2.55	1.28	0.68, 2.43
Student-teacher ratio					1.02	0.95, 1.09	1.02	0.95, 1.09
Intraclass Correlation (ICC)	0.0%		0.0%		0.0%			
Perceived problematic familial alcohol use Intermediate teacher-rated student focus*							*0.70 (p=0.354)*	*0.33, 1.49*
Perceived problematic familial alcohol use Strong teacher-rated student focus*							*0.22** (p=0.005)*	*0.08, 0.62*

***p<0.001 **p<0.01 *p<0.05

Model 1: Student level variables; Model 2: Student level variables + teacher-rated student focus; Model 3: Student-level variables + school segregation profile + student-teacher ratio; Model 4: Student level variables + teacher-rated student focus + school segregation profile + student-teacher ratio.

Supplementary Information. Table S4. Odds ratios (OR) and 95% confidence intervals (95% CI) from two-level binary logistic regressions of co-occurring somatic complaints among girls. n=3981 students in 147 senior-level school units. All models are adjusted for study year.

	Model 1		Model 2		Model 3		Model 4	
	OR	95% CI	OR	95% CI	OR	95% CI	OR	95% CI
Student level								
Perceived problematic familial alcohol use								
No (ref.)	1.00	-	1.00	-	1.00	-	1.00	-
Yes	1.47**	1.17, 1.86	1.47**	1.16, 1.85	1.50**	1.18, 1.89	1.49**	1.18, 1.88
Family structure								
Two-parent household (ref.)	1.00	-	1.00	-	1.00	-	1.00	-
Other	1.28**	1.07, 1.52	1.27**	1.06, 1.51	1.26*	1.06, 1.51	1.26*	1.06, 1.51
Parental university education								
No or not known (ref.)	1.00	-	1.00	-	1.00	-	1.00	-
At least one parent	0.98	0.82, 1.17	1.01	0.84, 1.20	1.03	0.86, 1.23	1.03	0.86, 1.24
Parental unemployment								
No parent unemployed (ref.)	1.00	-	1.00	-	1.00	-	1.00	-
At least one parent unemployed	1.64**	1.21, 2.22	1.64**	1.21, 2.22	1.61**	1.18, 2.18	1.62**	1.19, 2.19
Migration background								
≥10 years in Sweden (ref.)	1.00	-	1.00	-	1.00	-	1.00	-
<10 years in Sweden	0.84	0.61, 1.16	0.81	0.58, 1.11	0.77	0.56, 1.07	0.76	0.55, 1.06
School level								
Teacher-rated student focus								
Weak (ref.)			1.00				1.00	-
Intermediate			0.88	0.70, 1.11			0.94	0.74, 1.19
Strong			0.66**	0.52, 0.85			0.72*	0.55, 0.95
School segregation profile								
Privileged (ref.)					1.00	-	1.00	-
Typical					1.11	0.83, 1.48	0.96	0.71, 1.31
Deprived					1.44	0.99, 2.09	1.18	0.79, 1.77
Deprived immigrant					1.25	0.82, 1.90	1.06	0.69, 1.64
Student-teacher ratio					0.97	0.92, 1.02	0.97	0.93, 1.02
Intraclass Correlation (ICC)	3.3%		2.4%		2.5%		2.1%	
Perceived problematic familial alcohol use Intermediate teacher-rated student focus*							*0.88 (p=0.637)*	*0.51, 1.51*
Perceived problematic familial alcohol use Strong teacher-rated student focus*							*0.92 (p=0.774)*	*0.52, 1.64*

***p<0.001 **p<0.01 *p<0.05

Model 1: Student level variables; Model 2: Student level variables + teacher-rated student focus; Model 3: Student-level variables + school segregation profile + student-teacher ratio; Model 4: Student level variables + teacher-rated student focus + school segregation profile + student-teacher ratio.

Supplementary Information. Table S5. Unstandardised coefficients (b) and 95% confidence intervals (95% CI) from two-level linear regressions of psychological distress among boys. n=3963 students in 147 senior-level school units. All models are adjusted for study year.

	Model 1		Model 2		Model 3		Model 4	
	b	95% CI	b	95% CI	b	95% CI	b	95% CI
Student level								
Perceived problematic familial alcohol use								
No (ref.)	0.00	-	0.00	-	0.00	-	0.00	-
Yes	1.06***	0.82, 1.30	1.05***	0.81, 1.30	1.08***	0.83, 1.32	1.07***	0.83, 1.32
Family structure								
Two-parent household (ref.)	0.00	-	0.00	-	0.00	-	0.00	-
Other	0.24**	0.09, 0.40	0.24**	0.09, 0.39	0.24**	0.09, 0.39	0.24**	0.08, 0.39
Parental university education								
No or not known (ref.)	0.00	-	0.00	-	0.00	-	0.00	-
At least one parent	-0.21**	-0.36, -0.07	-0.21**	-0.36, -0.06	-0.18*	-0.33, -0.03	-0.18*	-0.33, -0.03
Parental unemployment								
No parent unemployed (ref.)	0.00	-	0.00	-	0.00	-	0.00	-
At least one parent unemployed	0.37*	0.04, 0.70	0.37*	0.04, 0.70	0.33	-0.00, 0.66	0.33	-0.00, 0.66
Migration background								
≥10 years in Sweden (ref.)	0.00	-	0.00	-	0.00	-	0.00	-
<10 years in Sweden	0.79***	0.53, 1.06	0.79***	0.53, 1.05	0.70***	0.43, 0.97	0.70***	0.42, 0.97
School level								
Teacher-rated student focus								
Weak (ref.)			1.00	-			0.00	-
Intermediate			0.04	-0.15, 0.24			0.10	-0.10, 0.30
Strong			-0.04	-0.25, 0.17			0.01	-0.21, 0.24
School segregation profile								
Privileged (ref.)					0.00	-	0.00	-
Typical					0.00	-0.23, 0.23	-0.01	-0.25, 0.24
Deprived					0.14	-0.18, 0.46	0.14	-0.20, 0.48
Deprived immigrant					0.48**	0.12, 0.84	0.48*	0.11, 0.86
Student-teacher ratio					0.03	-0.01, 0.07	0.03	-0.01, 0.07
Intraclass Correlation (ICC)	1.2%		1.1%		0.9%		0.8%	
Perceived problematic familial alcohol use Intermediate teacher-rated student focus*							*-0.67* (p=0.018)*	*-1.23, -0.12*
Perceived problematic familial alcohol use Strong teacher-rated student focus*							*-0.67* (p=0.035)*	*-1.30, -0.05*

***p<0.001 **p<0.01 *p<0.05

Model 1: Student level variables; Model 2: Student level variables + teacher-rated student focus; Model 3: Student-level variables + school segregation profile + student-teacher ratio; Model 4: Student level variables + teacher-rated student focus + school segregation profile + student-teacher ratio.

Supplementary Information. Table S6. Unstandardised coefficients (b) and 95% confidence intervals (95% CI) from two-level linear regressions of psychological distress among girls. n=3981 students in 147 senior-level school units. All models are adjusted for study year.

	Model 1		Model 2		Model 3		Model 4	
	b	95% CI	b	95% CI	b	95% CI	b	95% CI
Student level								
Perceived problematic familial alcohol use								
No (ref.)	0.00	-	0.00	-	0.00	-	0.00	-
Yes	1.15***	0.90, 1.41	1.15***	0.90, 1.41	1.14***	0.89, 1.39	1.14***	0.88, 1.39
Family structure								
Two-parent household (ref.)	0.00	-	0.00	-	0.00	-	0.00	-
Other	0.43***	0.25, 0.61	0.43***	0.25, 0.61	0.41***	0.23, 0.59	0.41***	0.23, 0.59
Parental university education								
No or not known (ref.)	0.00	-	0.00	-	0.00	-	0.00	-
At least one parent	-0.18*	-0.36, -0.01	-0.17	-0.34, 0.01	-0.15	-0.32, 0.03	-0.15	-0.33, 0.03
Parental unemployment								
No parent unemployed (ref.)	0.00	-	0.00	-	0.00	-	0.00	-
At least one parent unemployed	0.47**	0.13, 0.82	0.47**	0.12, 0.81	0.48**	0.13, 0.82	0.48**	0.13, 0.82
Migration background								
≥10 years in Sweden (ref.)	0.00	-	0.00	-	0.00	-	0.00	-
<10 years in Sweden	0.17	-0.15, 0.48	0.14	-0.17, 0.45	0.16	-0.16, 0.48	0.17	-0.16, 0.49
School level								
Teacher-rated student focus								
Weak (ref.)			0.00	-			0.00	-
Intermediate			-0.13	-0.36, 0.10			-0.12	-0.35, 0.12
Strong			-0.21	-0.45, 0.02			-0.05	-0.32, 0.21
School segregation profile								
Privileged (ref.)					0.00	-	0.00	-
Typical					0.44**	0.18, 0.70	0.43**	0.15, 0.72
Deprived					0.54**	0.18, 0.90	0.52*	0.12, 0.91
Deprived immigrant					0.18	-0.22, 0.58	0.14	-0.28, 0.56
Student-teacher ratio					-0.02	-0.07, 0.02	-0.02	-0.07, 0.02
Intraclass Correlation (ICC)	1.0%		0.9%		0.8%		0.8%	
Perceived problematic familial alcohol use Intermediate teacher-rated student focus*							*-0.53 (p=0.088)*	*-1.13, 0.08*
Perceived problematic familial alcohol use Strong teacher-rated student focus*							*-0.33 (p=0.301)*	*-0.94, 0.29*

***p<0.001 **p<0.01 *p<0.05

Model 1: Student level variables; Model 2: Student level variables + teacher-rated student focus; Model 3: Student-level variables + school segregation profile + student-teacher ratio; Model 4: Student level variables + teacher-rated student focus + school segregation profile + student-teacher ratio.
